# Automated Near Real‐Time QC for LC‐HRMS

**DOI:** 10.1002/rcm.70052

**Published:** 2026-02-17

**Authors:** Michael J. Mohr, Linus Strähle, Tobias Bader, Pia Leurle, Jan H. Christensen, Wolfram Seitz, Rudi Winzenbacher

**Affiliations:** ^1^ Zweckverband Landeswasserversorgung Stuttgart Germany; ^2^ Faculty for Plant and Environmental Sciences University of Copenhagen Copenhagen Denmark

**Keywords:** automated QA/QC, multivariate statistical process control, near real‐time analysis, online LC‐HRMS, QA/QC

## Abstract

**Rationale:**

The quality of analytical measurements is typically evaluated after completion of the entire, or possibly multiple, measurement batch(es). Automated, near real‐time quality control (QC) during LC‐HRMS acquisition can prevent reruns and sample loss by flagging issues as they occur. Functionality was evaluated by retrospective application to 5 years of river‐water surveillance.

**Methods:**

We present a modular MATLAB workflow that tracks isotopically labelled internal standards for peak height, retention time and mass error against rolling, method‐specific expectations; applies multivariate statistical process control (MSPC; PCA with Hotelling's *T* ^2^ and SPE on intensity/retention time ratios and mass error); issues immediate email alerts; and logs outcomes to a PostgreSQL database/Grafana dashboard for trend analysis. Also, qualitative target screening via cosine‐similarity MS^2^ checks against a local library, retention time correction, robust peak‐height/noise estimation, configurable limits and automated vendor‐to‐open format conversion.

**Results:**

In a high‐voltage power‐supply failure, 25/25 injections were flagged due to abnormal intensity patterns; during an organic‐pump malfunction, 17/25 were flagged for retention drift up to and beyond the extraction window; and during an air‐conditioning (AC) outage, MSPC detected mass error anomalies even when the ±10 ppm univariate limit was not breached. MSPC closely agreed with univariate thresholds: 95.7% of samples flagged by univariate rules were also flagged by MSPC (≈4.3% Type II), while 92.5% of MSPC‐flagged samples violated at least one univariate rule (≈7.5% Type I).

**Conclusion:**

These capabilities enable immediate detection, triage and documentation of performance excursions, support proactive maintenance (e.g., column aging or pump delivery issues), minimise downtime and safeguard precious samples. Although showcased on a specific LC‐HRMS setup and matrix, the workflow is instrument‐agnostic and broadly applicable to internal‐standardised LC‐HRMS methods.

## 1 Introduction

Liquid chromatography–mass spectrometry (LC‐MS) is the gold standard for trace analysis of non‐volatile organic compounds across food safety, pharmaceuticals, clinical diagnostics, forensic science and environmental monitoring [1, 2]. While low‐resolution MS is typically used for targeted detection of predefined substances [3], high‐resolution instruments (LC‐HRMS) improve on three key aspects:

(1) Broad‐spectrum screening—simultaneous detection of hundreds to thousands of compounds in a single run

(2) Identification of unknowns—discovery and investigation of previously unknown or unexpected substances

(3) Retrospective analysis—re‐evaluation of stored data for newly prioritised compounds

Despite increasing automation in LC‐(HR)MS hardware and software [4], data evaluation often remains manual, creating delays between measurement and interpretation. To improve reliability and avoid costly reworks (reruns and data re‐evaluation), it is essential that users monitor system performance to ensure measurements stay within specifications. Time‐consuming and repetitive, routine quality control (QC) workflows for targeted analysis have been largely standardised (e.g., DIN EN ISO/IEC 17025:2017). This is not the case for non‐target screening, where QC procedures are less standardised. Previous suggestions in that area have been criticised for their focus on reducing false‐positive (Type I error) [5, 6] and neglect of false‐negative (Type II Error) identifications. For both approaches, there is a significant gap between occurrence and reporting [7], since QC is mostly performed after the measurement. Near real‐time process monitoring addresses this gap. For QTOF instruments, the regular calibration files can be utilised to assess instrumental performance in near real‐time [8]. However, this does not cover the performance of the (chromatographic) separation. Also, the frequency of calibration files is usually limited (e.g., one file every 2 h). Workflows for automated QC based on recently measured samples have also been devised [9], but they are specific to a type of data (e.g., proteomics), thus preventing their application in other fields. In this work, we present an instrument‐agnostic and broadly applicable workflow for near real‐time QC: *Automated Quality Control for Mass Spectrometry (AutoQ4MS)*. In *AutoQ4MS*, the quality of each measurement is assessed by continuously comparing key QC metrics such as internal standard retention time, peak intensity and mass error to recent measurements. The same metrics can be tracked for target analytes in real samples with minimal added effort, enabling rapid qualitative assessment of sample composition. Target identifications can be further supported by comparison with MS^2^ spectral libraries. Results are stored in a structured database, facilitating long‐term monitoring, cross‐batch comparison and statistical evaluation, particularly in large studies or for real‐time assessments. This work evaluates near‐real‐time process control using *AutoQ4MS*, a GUI (graphical user interface)–based application that performs continuous near real‐time QC and warning, with automatic database upload and dashboard visualisation for long time‐series. We demonstrate performance on 5 years of river‐water surveillance data, with special emphasis on instances that require reruns due to instrument issues, illustrating how near‐real‐time monitoring could have prevented delays. *AutoQ4MS* can be downloaded from GitHub (https://github.com/Landeswasserversorgung/AutoQ4MS).

## 2 Materials and Methods

### 2.1 Chemicals and Standards

All measurements used LC‐MS grade acetonitrile (ACN) and ultrapure LC‐MS grade water. Formic acid (0.1%, purity > 99% in LC‐MS grade) was added to both solvents.

### 2.2 Instrumental Analysis

The LC‐HRMS acquisition method is described in [10]. Separations were performed on an LC20 series system (Shimadzu, Kyoto, Japan) using a Zorbax Eclipse C18 plus column (150 × 2.1 mm, 3.5 μm; Agilent, Santa Clara, United States) at 40°C. Samples (95 μL) were co‐injected with 5 μL of internal standard solution via the autosampler. The LC was coupled to a TripleTOF 5600 QTOF (Sciex, Framingham, United States). Acquisition was performed with MS^1^ (m/z 100–1200), data‐dependent (DDA/Top10) and data‐independent (DIA/SWATH) MS^2^ acquisition. Resolving power ranged from 23 000 at m/z 146.08834 to 36 000 at m/z 922.00980 (as of 20 January 2025).

### 2.3 Data Processing Workflow

**FIGURE 1:**
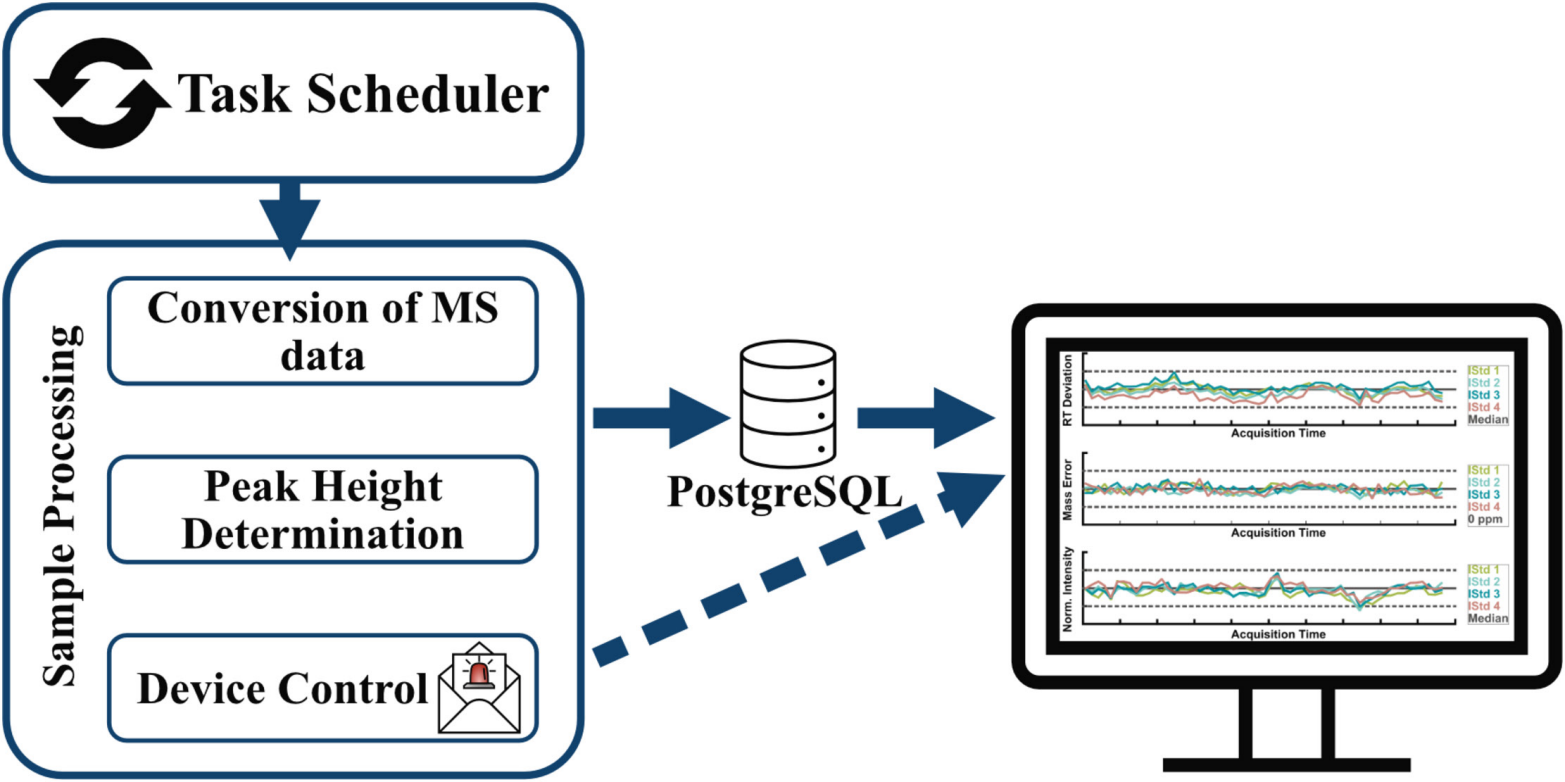
Schematic workflow of AutoQ4MS. A compound list (internal standards and targets) with expected retention times and exact m/z was stored in the program folder. AutoQ4MS periodically checks a specific folder (including subfolders) for unprocessed MS data and performs automated QC.

*AutoQ4MS* (MATLAB 2024; MathWorks, Natick, United States) served as the central QC pipeline (Figure 1). Method‐specific parameters are configurable via a GUI. A Windows Task Scheduler job triggered the sample processing script at regular intervals, found newly completed LC‐HRMS files, and, after a ≥ 2 min no‐modification window, attempted conversion/loading of each unprocessed dataset. Vendor files (e.g., *.wiff, *.wiff2, *.raw) were converted to open *.mzXML using MSConvert [11], then imported into MATLAB [12] for peak‐height determination, MS^2^ comparison and quality‐control checks. Files that fail to convert automatically are skipped. In the next iteration, conversion is reattempted. Results were reported to PostgreSQL (postgresql.org) and visualised in Grafana (grafana.com), both hosted on a Linux server.

#### 2.3.1 Retention Time Correction

To address long‐term retention time drift (e.g., column ageing/replacement) [13, 14], stored retention times were corrected by the median retention time deviation of all internal standards in the most recent blank. In blanks, the retention time tolerance for internal standards was tripled relative to regular samples to allow retention time correction beyond the tolerances defined for peak‐picking.

#### 2.3.2 Peak‐Height Determination

**FIGURE 2:**
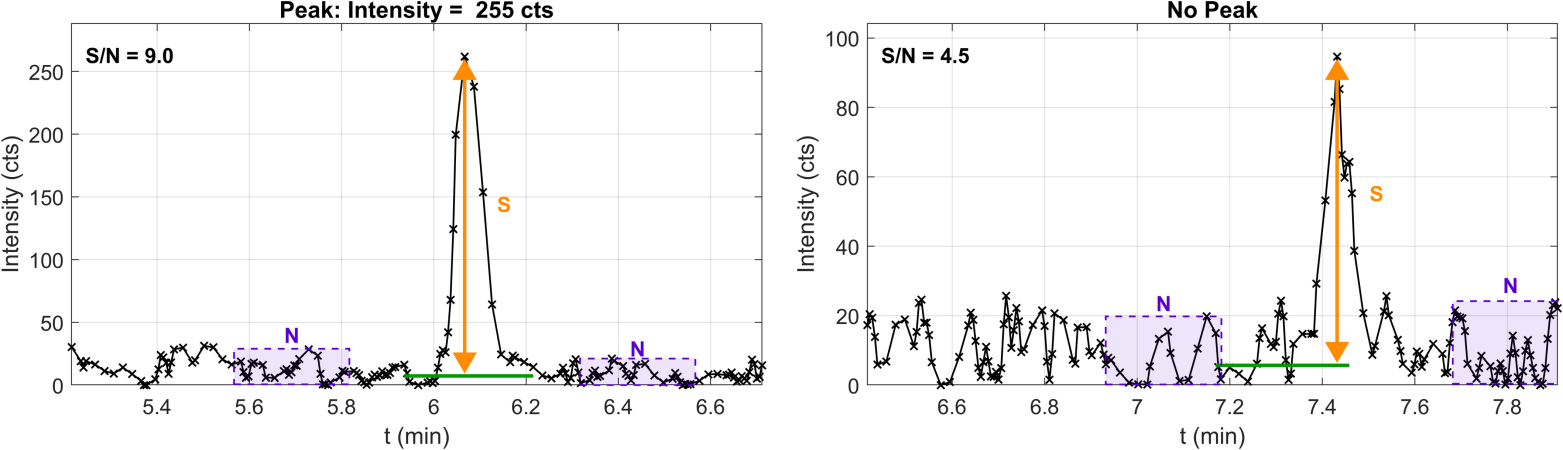
Peak‐height determination in the EIC. Baseline and expected retention time shown as horizontal green lines; signal ‘S’; noise windows ‘N’.

Since peaks were detected without the possibility of human intervention, peak height was chosen instead of peak area, which would usually be preferable. Peak height is more robust mathematically, and, therefore, reduces the risk of causing alarms due to inaccurate peak detection (i.e., integration of peak area). For each compound, an extracted‐ion chromatogram (EIC) was generated at exact mass ±20 ppm. The extraction of such a large mass window allowed detection of mass errors above the defined limit of ±10 ppm. A ± 9 s retention time window centred on the apex was extracted and smoothed twice with a Savitsky–Golay filter (quadratic, five‐point window) [15]. Two 15 s noise windows were taken: one starting 30 s before the peak, one starting 15 s after (labelled ‘N’). The baseline (green line below the peaks) was the mean of the lower‐noise window. Noise was estimated as (max–min) within each noise window; the smaller value was used to avoid counting isobaric co‐elution as noise. Peaks were accepted if both criteria held: (i) S/*N* > 5 and (ii) ≥ 5 EIC points above (baseline +2 × noise). See Figure 2.

#### 2.3.3 Device Control

Instrument performance was assessed using normalised peak height, retention time and mass error of isotopically labelled internal standards. The normalised intensity of each internal standard was calculated against its median signal over the past 60 days. Absolute retention time deviation was the difference from the 60‐day median. Mass error was the ppm deviation of the mean m/z from the five spectra around the apex versus the theoretical m/z.

Limits (empirically set from > 5 years on the same instrument/method/matrix) were as follows:

(1) Normalised intensity: 0.5–2.0

(2) Mass error: ±10 ppm

(3) Retention time deviation: ±10 s

Internal standard parameters of the 3352 measurements can be found in Supporting Information: ‘Internal Standard Table‐data‐2026‐01‐07 08_44_41.csv’. Limits are system‐/method‐dependent and configurable. Since the mass error of QTOF instruments results from the measurement uncertainty of the ion's flight‐time, it is approximately constant over the m/z range. While mass errors of below 2 ppm are generally achievable using QTOF instruments [16], this is not necessarily the case for small masses (i.e., Benzothiazole‐d4, m/z 124.0807, 10 ppm = 1.24 mDa). Despite the relative mass error (in ppm) being commonly used for QTOF instruments, the absolute mass error (in mDa) might be more consistent over the m/z range. If any internal standard breached a limit, an entry was logged, and an immediate email was sent with time‐history plots and issue details (example in ‘SI of Automated near real‐time QC for LC‐HRMS.docx’).

##### 2.3.3.1 Target MS2 Check

For targets where DDA spectra were acquired, *AutoQ4MS* computed cosine similarity to a local library (*.json per compound), created from standards via the GUI ‘MS^2^ Library’ button. The database stored the acquired MS^2^ spectrum, number of matching fragments (± mass tolerance) and binned (e.g., 0.5 Da) cosine similarity (dot product between two non‐zero vectors). External libraries can be used if *.json filenames match the unique identifiers, and they adhere to the naming template (see included library).

##### 2.3.3.2 Multivariate Statistical Process Control (MSPC)

**FIGURE 3:**
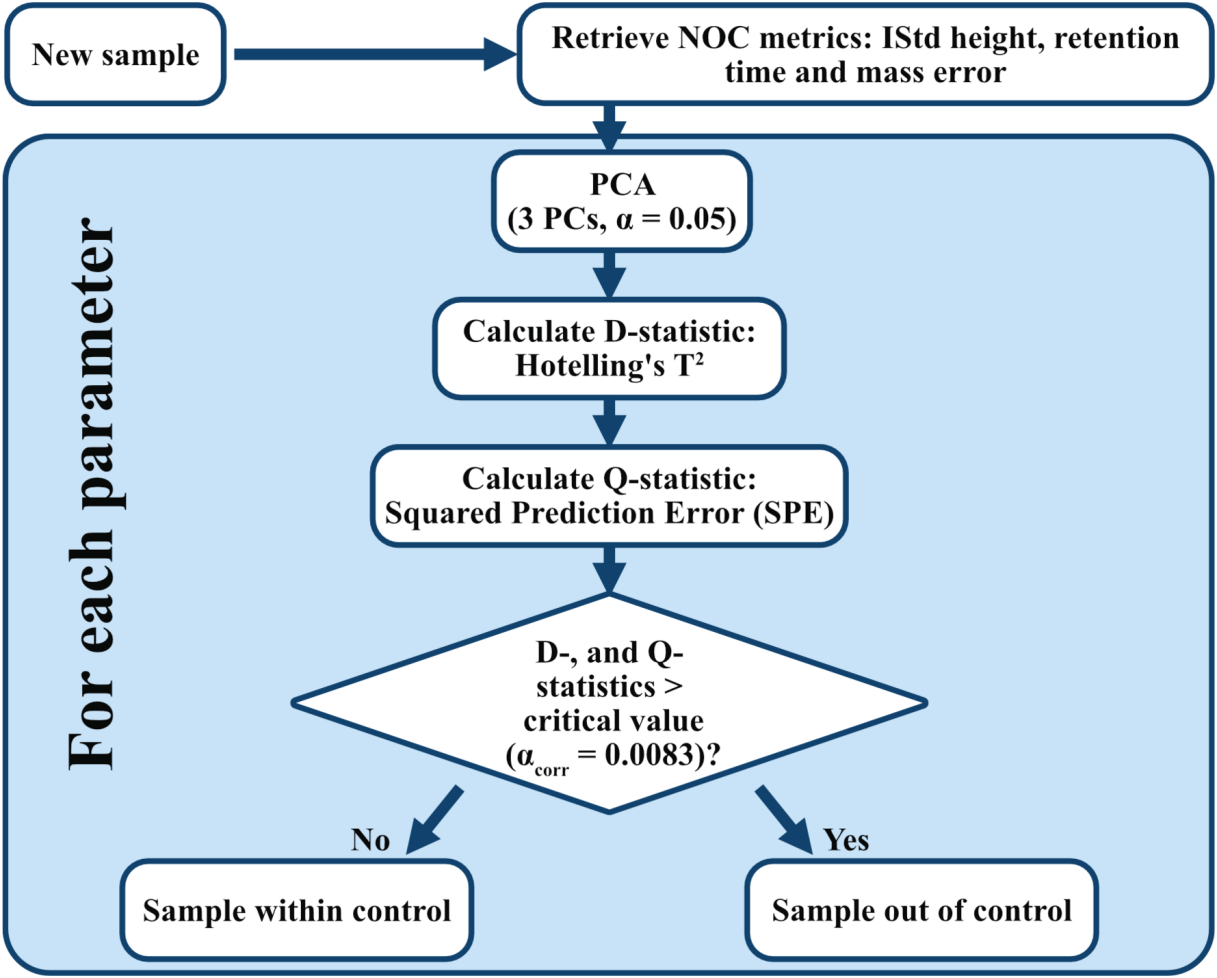
Overview of MSPC workflow. Only samples that passed (univariate) QC are considered as ‘normal operating conditions’.

We evaluated MSPC following the common practice for industrial batch processes [17, 18] (Figure 3). For each new sample, internal standard height, retention time and mass error from QC‐passing samples over the past 60 days were retrieved. To reduce systematic shifts (e.g., detector fluctuations) [19], ratios were formed for height and retention time for all unique internal standard pairs per Equation (1).

Equation 1 Ratios of internal standard (IStd) height and retention time.

$$\begin{array}{c} R_{\text{IStd}1/\left(\text{IStd}1+\text{IStd}2\right)}=\frac{{\text{IStd}}_{1}}{{\text{IStd}}_{1}+{\text{IStd}}_{2}}\\ R=\text{ratio};\text{IStd}=\text{internal standard}\;\left(\text{height}/\text{retention time}\right) \end{array}$$

These peak tables defined normal operating conditions (NOC). Separate PCAs were built for height, retention time and mass error. Each new sample was then projected onto said PCAs. Missing raw values (i.e., not detected internal standards) of the new sample were set to zero before autoscaling. Instead of imputation (e.g., of zero or the median), more sophisticated strategies for handling missing data are described in the literature [20]. However, imputation of zeroes was sufficient for this application, as it resulted in the sample being flagged for anomalies in peak height and retention time. Samples where any internal standard was not detected were excluded from NOC via a minimum number of required internal standards per sample and polarity. Therefore, it was not possible for this imputation of zeroes to affect the NOC. In PCA space, *Hotelling's T* ^*2*^ [21] (D‐statistic) tested for systematic bias; the squared prediction error (SPE) tested reconstruction fidelity. Here, the first three PCs were used at *α*_initial_ = 0.05. A Bonferroni correction [22] for six tests per sample gave a final *α*_corrected_ = 0.00833 (Equation (2)). The three principal components resulted in a median explained variance of 80.5%–85.3% (depending on parameter and polarity), with a minimum of 60%.

Equation 2 Bonferroni correction.

$$\begin{array}{c} \alpha_{\text{corrected}}=\frac{\alpha_{\text{initial}}}{n}\alpha =\text{level of significance};\\ n=\text{number of simultaneous hypothesis tests} \end{array}$$

Samples where both *T* ^2^ and SPE for any parameter (height, retention time or mass error) rejected the null hypothesis (H_0_) were flagged ‘out of control’. Variable contributions were computed as score × loading [23, 24]. Due to a lack of a quantifiable ground truth, the results of the univariate thresholds were used to estimate error rates of MSPC. Relative to univariate thresholds, 95.7% of univariate‐flagged samples were also MSPC‐flagged (≈4.3% Type II), while 92.5% of MSPC‐flagged samples violated ≥ 1 univariate rule (≈7.5% Type I).

Compared to fixed thresholds, MSPC offers the following:

1. Minimal method‐specific tuning (default α and a small number of PCs often suffice).
2. Limits derived from the distribution and variance of recent in‐control samples, which passed previous QC checks.
3. Correlations between internal standards are considered. For example, systematic retention time shifts of several internal standards indicate column ageing, while peak inversions (i.e., change of elution order) suggest severe deterioration [13, 14].

Limitations to note:

1. *T* ^2^ limits can become extremely large with small sample sizes.
2. Exceedances are less directly interpretable; translating a multivariate alarm to the magnitude of change in a single metric (e.g., mass error) is not straightforward.

## 3 Results and Discussion

### 3.1 Entire Data Set

**FIGURE 4:**
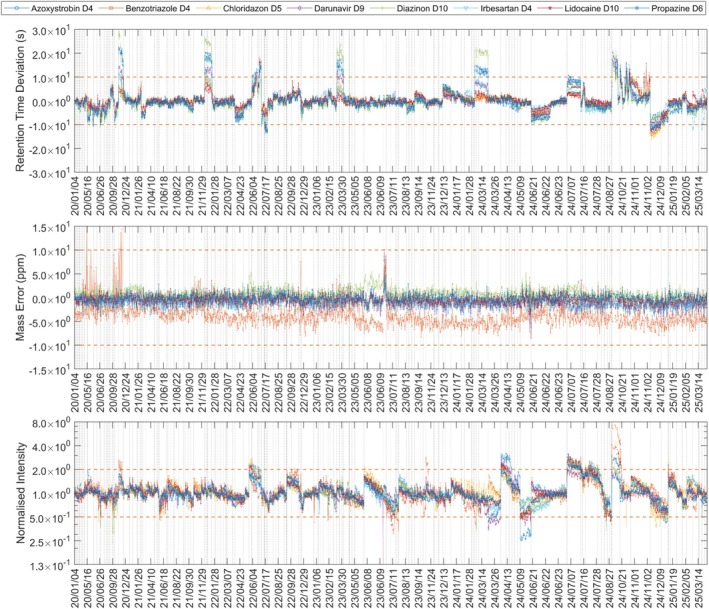
Retention time deviation, mass error and normalised intensity for eight internal standards in raw‐water surveys (ESI+), 1 January 2020–23 April 2025. Horizontal dashed lines indicate control limits; vertical dashed lines mark batch boundaries. The *x*‐axis is discontinuous; periods without acquisition are omitted.

Since 2018, the Landeswasserversorgung has routinely been monitoring organic micropollutants in the Danube using LC‐HRMS. *AutoQ4MS* performance and its capability to flag instrumental issues were assessed via a retrospective analysis of raw‐water surveys from January 2020 to April 2025. Fourteen batches (495 injections) with known performance problems were included. Figure 4 summarises all detected deviations in ESI+. Of the 3352 measurements (ESI+ and ESI−), 739 were in violation of at least one univariate threshold (e.g., normalised intensity 0.5–2.0; retention time ±10 s; mass error ±10 ppm). For 125 measurements, at least one internal standard was not detected. Of the remaining 614 anormal measurements, 370 were flagged due to peak height, 272 due to retention time, 6 due to mass error and 34 due to multiple reasons.

### 3.2 Examples for Instrument Performance Issues

**FIGURE 5:**
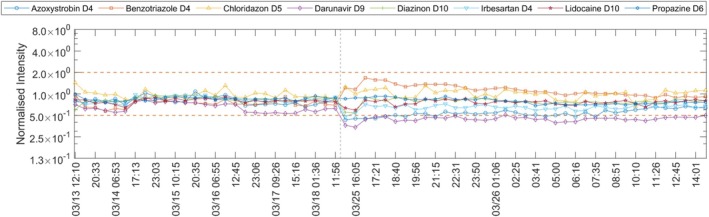
Normalised intensity of eight internal standards (ESI+) during the raw‐water survey between 13 and 26 March 2024. Periods without acquisition are omitted.

**FIGURE 6:**
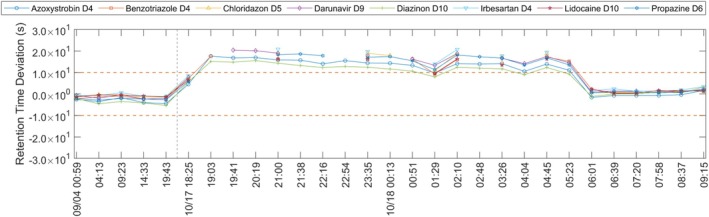
Retention time deviation of eight internal standards (ESI+), 4 Sep–18 Oct 2024; periods without acquisition omitted. Missing points indicate shifts beyond the 27 s extraction window.

**FIGURE 7:**
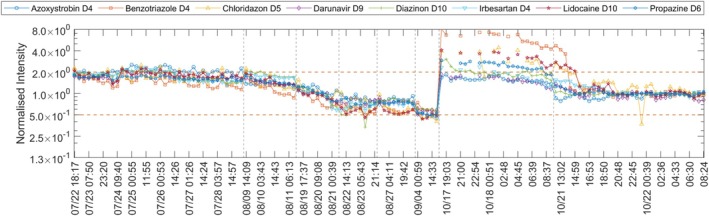
Normalised intensity of eight internal standards (ESI+) during the raw‐water survey between 22 June 2024 and 22 October 2024. Horizontal dashed lines denote control limits; vertical dashed lines mark batch boundaries. The *x*‐axis is discontinuous (periods without acquisition removed).

**FIGURE 8:**
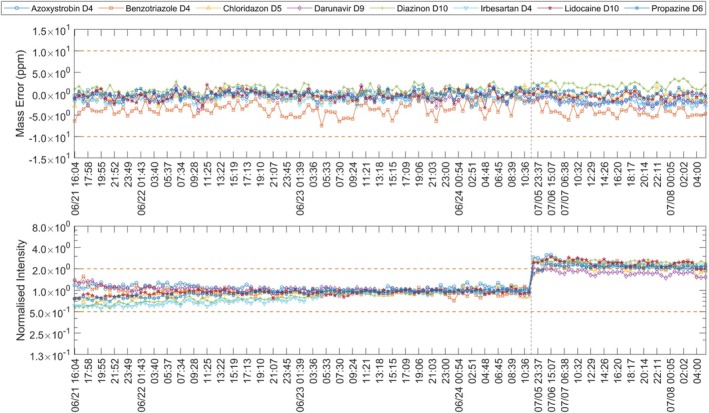
Mass error (top) and normalised intensity (bottom) of eight internal standards (ESI+), 21 June—8 July 2024. Horizontal dashed lines show control limits; the vertical dashed line marks the start of a new batch. Periods without acquisition are omitted.

Instrumental problems are often detected only during post‐acquisition review. By then, defects may have persisted for days, wasting time and samples that cannot be recollected. Within the river‐water surveillance program, such events occurred annually. Four representative cases are detailed below in Figures 5, 6, 7, and 8.

#### 3.2.1 Normalised Intensity 2024 March 25th–26th

On 25 March, two internal standards, Darunavir‐d9 (m/z 557.299, 10.41 min) and Azoxystrobin‐d4 (m/z 408.1492, 11.80 min), showed a sharp intensity loss, whereas Benzotriazole‐d4 (m/z 124.0807, 5.56 min) and Chloridazon‐d5 (m/z 227.0743, 6.50 min) increased (Figure 5). Across all internal standards, signals for compounds with higher m/z than Propazine‐d6 (m/z 236.1544, 11.00 min) decreased in at least the first two injections of the batch, with larger effects at higher m/z (i.e., Irbesartan‐d4, m/z 433.2648, 8.87 min) than the lower m/z (i.e., Lidocaine‐d10, m/z 245.2433, 5.31 min). No relationship with retention time was evident. AutoQ4MS analysis would have flagged the issue in real time (25/25 warnings for 25 injections). If the acquisition had been stopped after the first warning, this could have prevented 12 h of wasted run time and sample consumption. Cleaning the ion source and retuning the QTOF did not resolve the problem. Two days later, the vendor software reported ‘HV supply error’ and ‘pulser module error’; replacing the HV power supply resolved the fault.

#### 3.2.2 Retention Time Deviation 2024 October 17th—18th

Retention time shifts of up to the 27 s window limit were observed; some internal standards exceeded this window and were not detected. In retrospect, *AutoQ4MS* issued warnings for 17 of 25 injections (visualised in Figure 6). Diazinon‐d10 (m/z 315.1711, 14.10 min) and Azoxystrobin‐d4 (m/z 408.1492, 11.80 min) were slightly less affected. In ESI+, they were the latest‐eluting standards. The reversed‐phase gradient reached 100% organic at 16.50 min and held until 27 min. Retention times shifted to later times across the batch, most prominent for early‐eluting internal standards such as Lidocaine‐d10 (m/z 245.2433, 5.31 min). Most likely, this was due to insufficient solvent delivery by the organic‐phase pump. This diagnosis was supported by the next run: the organic‐pump piston‐lubricant reservoir (Milli‐Q/10% isopropanol, v/v) had filled, indicating the pump was not drawing solvent properly, possibly due to defective plunger seals. If *AutoQ4MS* had been available at that time, the issue would have been flagged immediately. Instead, an additional batch was acquired before the evaluation and detection of the fault. In addition to the retention time shift, the batch showed markedly elevated internal standard intensities compared to the 60‐day median, most pronounced for early‐eluting, low‐mass internal standards (Benzothiazole‐d4, m/z 124.0807, 5.56 min; Lidocaine‐d10, m/z 245.2433, 5.31 min; Chloridazon‐d5, m/z 227.0743, 6.50 min). As this batch was the first run after the annual QTOF maintenance, which corrected a steady intensity decline present since 9 August 2024, the elevated intensity was attributable to maintenance. Figure 7 shows the corresponding normalised intensities; as in Figure 6, line breaks occur where peaks are shifted outside the expected retention time window.

#### 3.2.3 Mass Error Deviation 2024 July 5th—8th

An instrumental warning was issued for all 34 measurements. As Figure 8 shows that most univariate limit breaches in the second batch (right of the vertical line) stem from poor signal in the preceding measurements: after cleaning the ion transfer, intensities rose and fell outside the previously calculated boundaries. Mass error was slightly higher than usual but remained within ±10 ppm, as can be seen in Figure 8. Retention times shifted by 5–10 s for most standards; the ±10 s limit was exceeded twice.

**FIGURE 9:**
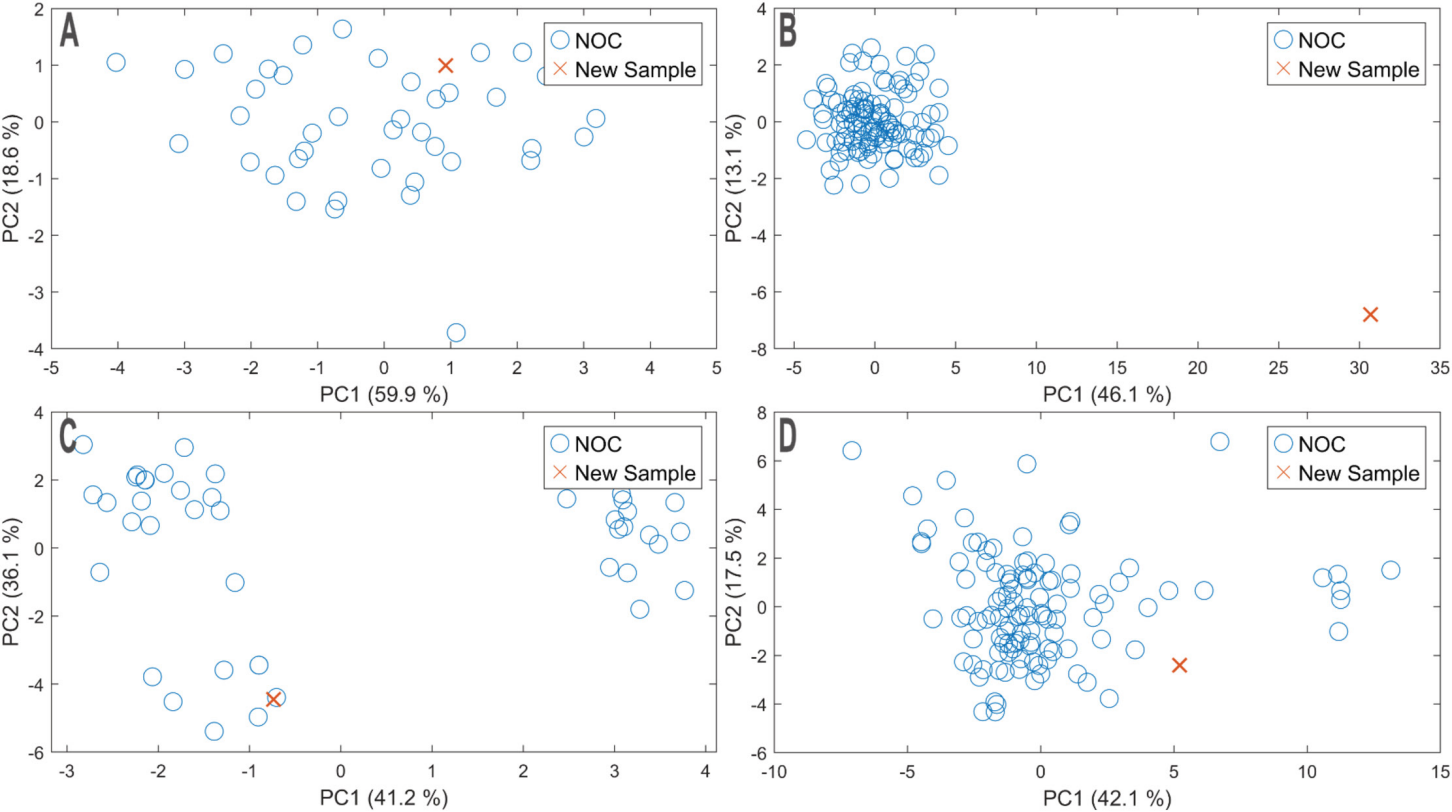
PCA score plot for internal standard mass errors. Left: last sample within NOC. Right: first sample ‘out of control’. As described in the Materials and Methods section, PCA was built on NOC samples only; new samples were projected onto this model, so the explained variance (%) is computed without the new sample.

However, MSPC identified mass error anomalies in 33 of 34 measurements, even though the ±10 ppm limit was never crossed. The remaining run was a 0 injection and was flagged for peak height and retention time; its mass error was treated as 0 ppm. In total, five measurements were flagged by MSPC due to intensity and two due to retention time. Visual inspection of the mass errors suggested little difference between the prior batch (22 June 2024 to 25 June 2024) and the current batch. Yet, the second batch (7 July 2024 to 8 July 2024) showed median/mean mass errors up to 1.3 ppm farther from zero for some standards, too small for the univariate alarm, but evident in PCA scores (Figure 9). Here, mass error produced a clear shift on PC1–PC2: the last in‐control sample (Figure 9A) lay within the NOC cluster, while the first sample of the new batch (B) projected well outside. Both D‐ and Q‐statistics rejected H_0_ for mass error data. PC3 showed no effect and is omitted. For internal standard intensity ratios (also Figure 9), both samples (Figure 9C,D) fell within the NOC cluster; *T* ^2^ (D‐statistic) did not reject H_0_. The first sample of the new batch (Figure 9D), however, rejected H_0_ for the Q‐statistic, indicating increased residual variance (noise), not explained by the NOC PCA model. This matches Figure 8 (bottom): intensities increased after cleaning, but not by the same factor for all internal standards. This shift resulted in an increased prediction error because the NOC model encoded different internal standard ratios.

MSPC rarely issued warnings because of systematic shifts in the internal standard intensities (i.e., after cleaning/maintenance) compared to fixed thresholds. Likely because it operated on ratios of internal standards and not absolute values. This made the shift nearly invisible when all internal standards shifted by a similar factor. Ratios could also be used for univariate thresholds. However, the purpose of the univariate thresholds was to detect such systematic shifts compared to the median of recent measurements. Furthermore, the MSPC was able to detect anomalies in mass error, even though the threshold was not exceeded. The anomaly pointed to a root cause: on 8 July 2024, the lab's air‐conditioning (AC) had failed over the weekend, resulting in a room temperature > 30°C. Two other HRMS interrupted their measurement due to the temperature. Calibration files showed larger mass errors; the QTOF's internal mass correction (background‐ion lock masses at run start, as on SCIEX systems) largely compensated during acquisition, but not fully. Consequently, MSPC flagged every measurement with internal standards in the second batch due to mass error anomalies. An increase of mass errors by 1.3 ppm is neither critical for evaluation nor should it trigger a warning immediately. However, tracking such anomalies as they progress over time can enable users to detect faults before a major issue occurs. Thereby, allowing preventive maintenance to be scheduled at a convenient time. Such a trend analysis could be based on tracking the thresholds and calculated values of the statistical tests (Q‐ and D‐statistics). Both, as well as a list of affected samples, are uploaded to the database. For example, if each measurement of a batch is flagged for retention time anomalies, but the threshold of ±10 s is not exceeded, consumables in the HPLC can be replaced during the next maintenance.

### 3.3 Influence of the Bonferroni Correction on MSPC

A significant overlap between warnings generated by the univariate thresholds and MSPC (≈94%) was observed. This could partially be attributed to the Bonferroni correction during MSPC, reducing the α level (0.05 ➔ 0.00833) and thereby the likelihood to reject H_0_. Removal of this correction would have resulted in a lower threshold and thereby a larger number of issued warnings as well as an increased ratio of false‐positive (Type I) rejections of H_0_. For large numbers of hypothesis tests, the Bonferroni correction can result in an α level too low to detect true positives. In such cases, more gentle correction models, such as the false discovery rate [25], might be more suitable. For six hypothesis tests, the differences between these corrections can be neglected in most cases.

## 4 Conclusion

*AutoQ4MS* detected deviations from expected instrumental performance and issued warnings, often on the first sample. Faulty runs could have been stopped early, saving time and sample volume. A critical advantage, if samples are difficult to recreate or are expected to degenerate over time. Because the dataset included batches with known anomalies, warnings also appeared when internal standard signals returned to normal levels after a prolonged decline (post‐maintenance). The upper intensity limit guards against detector saturation. Nevertheless, responses to alerts should be system‐specific. After planned maintenance, a temporary increase of the upper limit can reduce unnecessary warnings. In one example (retention time shift), the likely cause of the shift in retention times could be narrowed down by considering which internal standards were most affected. Given enough experience, it might be possible to find many such correlations and prioritise a small number of likely causes for the fault in the troubleshooting process. Some issues were not captured. Lab staff noted problems that *AutoQ4MS* did not flag (i.e., high blank values and elevated TIC), since they were not included in the automated QC workflow. In other cases (i.e., mass error deviation), systematic shifts in internal standard peak parameters were observed, but because fixed thresholds were not crossed, no univariate warnings were issued. However, this shift was captured by MSPC. Both options (uni‐ and multivariate QC) have unique advantages. The fixed thresholds offer predefined quality criteria and a robust and straightforward assessment of the measurement's quality. In contrast, multivariate statistical process control is based on the actual mean and variance of recent measurements. Therefore, it can detect anomalies, which are not easily observable using multiple univariate thresholds. Thereby, it can contribute to detecting faults before serious damage is caused. Anomalies, for example, in retention time, might be caused by parts of the LC approaching the end of their lifetime. In such cases, the stockpile of spare parts could be replenished in time for maintenance to be scheduled, thus avoiding downtime of the instrument. However, as shown in the last example, MSPC detecting anomalies in the measurement does not necessarily mean that it cannot be evaluated. The bias in the mass errors, while statistically significant, was small enough to allow evaluation as usual. Still, it hinted at an underlying fault: the defective AC. Therefore, the actual values resulting in the issued warning, which are also shown on the dashboard and included in any emails, should always be considered before making any decisions.

## Author Contributions

**Michael J. Mohr:** writing – original draft (lead), formal analysis (lead), investigation, software (supporting); visualisation; validation, methodology (lead), writing – review and editing. **Linus Strähle:** software (lead), writing – original draft, visualisation (supporting), writing – review and editing. **Tobias Bader:** conceptualisation (lead), supervision, writing – review and editing, validation. **Pia Leurle:** investigation, data curation, validation. **Jan H. Christensen:** supervision, writing – review and editing (lead), methodology. **Wolfram Seitz:** writing – review and editing, conceptualisation. **Rudi Winzenbacher:** funding acquisition, conceptualisation, supervision.

## Funding

This work was supported by Zweckverband Landeswasserversorgung.

## Conflicts of Interest

The authors declare no conflicts of interest.

## Supporting information




**Figure S1:** Example plot of email warning. For the last two measurements, 1/5/3× of the usual amount of internal standard was co‐injected to test generation of warnings.

## Data Availability

The data that support the findings of this study are available from the corresponding author upon reasonable request.
