# Supplementary material for: Automated Near Real‐Time QC for LC‐HRMS
Source: Rapid Commun Mass Spectrom. 2026 Feb 17;40(10):e70052. doi: 10.1002/rcm.70052 (PMC12933221; doi:10.1002/rcm.70052)
Supplement: Supplementary file 1 — Figure S1: Example plot of email warning. For the last two measurements, 1/5/3× of the usual amount of internal standard was co‐injected to test generation of warnings. [file RCM-40-e70052-s001.docx]

# SI of Automated near real-time QC for LCHRMS

## Example Email

*AutoQ4MS* automatically tracks retention time, mass error and peak intensity of all specified internal standards and compares retention time and intensity to an x-day median. The warnings were tested by using 1/5 and 3 times the amount of internal standard (last two injections). Upon detection of a threshold violation, an email is sent to specified users, containing metadata as well as a time-history plot (Figure 1).


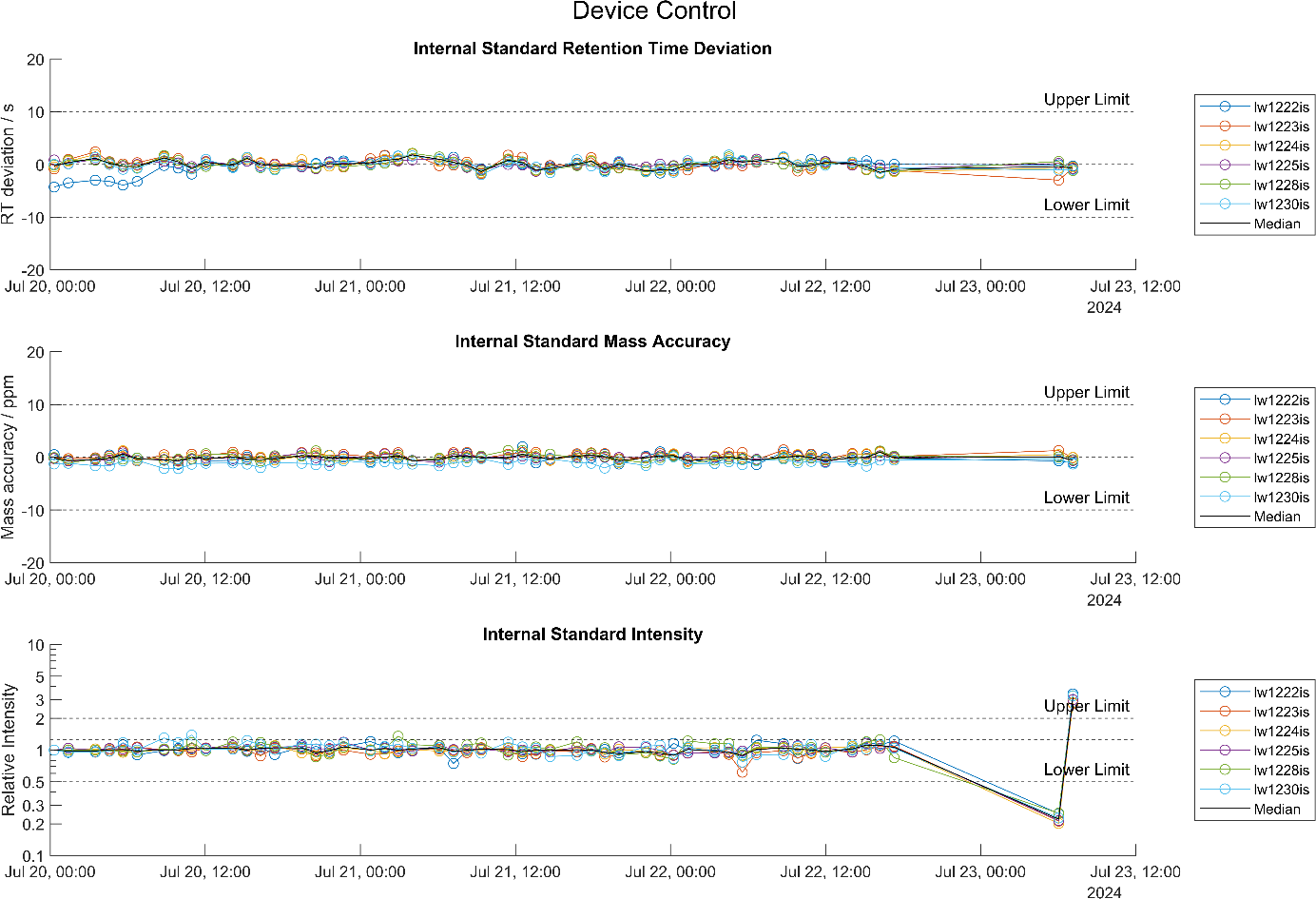


Figure 1 Example plot of email warning, For the last two measurements, 1/5 / 3x of the usual amount of internal standard was co-injected to test generation of warnings.
